# Predictive factors for refractory stage I and II anti-resorptive agent-related osteonecrosis of the jaw

**DOI:** 10.1007/s11282-021-00547-1

**Published:** 2021-07-03

**Authors:** Takahiro Shimizu, Mai Kim, Trang Thuy Dam, Jun Kurihara, Masaru Ogawa, Takaya Makiguchi, Satoshi Yokoo

**Affiliations:** 1 Department of Oral and Maxillofacial Surgery, and Plastic Surgery, Gunma University Graduate School of Medicine, 3-39-22, Showa-machi, Maebashi, Gunma 371-8511 Japan; 2 Department of Diagnostic Radiology and Nuclear Medicine, Gunma University Graduate School of Medicine, 3-39-22, Showa-machi, Maebashi, Gunma 371-8511 Japan

**Keywords:** ARONJ, BMA administration period, One scintigraphy, PET, CT, Refractory factor

## Abstract

**Objectives:**

We aimed to predict the possibility of patients with stage I and II anti-resorptive agent-related osteonecrosis of the jaw (ARONJ) developing resistance to our treatment protocol by evaluating their clinical and imaging factors.

**Materials and methods:**

We enrolled 58 patients with ARONJ who underwent imaging modality. As objective variables, we considered the healing, stage-down, and stable stages as successful outcomes, and the stage-up stage as resistant-to-treatment. As explanatory variables, we investigated the clinical and imaging factors. Furthermore, we examined stage-down as an improvement outcome to compare with the stable and stage-up stages, which were considered as no-improvement outcomes. We conducted unpaired between-group comparisons on all explanatory variables using *χ*^2^ tests for independence.

**Results:**

Among 58 patients, the treatment was successful in 53 (91.4%); however, the disease was resistant in five (8.6%). Among the clinical factors, the resistant patients had a longer duration of administration of bone-modifying agents (BMAs) (cut-off: 1251 days, *p* = 0.032, odds ratio = 11.2, 95% confidence interval 1.115–122.518). In addition, the target disease that was being treated bone metastasis of malignant tumor was the only significant refractory factor (*p* = 0.024, OR: 3.667 95% CI 1.159–11.603)

**Conclusions:**

A combination of metabolic and morphological imaging modalities may be useful for oral surgeons to evaluate the disease activity and predict course of refractory ARONJ.

## Introduction

Several imaging modalities and analytical methods have been used to assess the course of anti-resorptive agent-related osteonecrosis of the jaw (ARONJ) and determine its clinical stages [1]. According to the Japanese Society for Oral and Maxillofacial Radiology [2], 64% of oral radiologists believe that dental panoramic radiography (DPR) is the best screening modality for bisphosphonate-related osteonecrosis of the jaw (BRONJ), while 68% of oral radiologists prefer intraoral radiography, as it enables clear visualization of the trabecular bone structure around the tooth and the surrounding alveolar processes. Intra-oral radiography and DPR can be used to diagnose ARONJ in the oral or maxillofacial regions of asymptomatic patients treated with low-dose bone-modifying agents (BMAs) [2]. Computed tomography (CT) and dental cone-beam CT are effective in patients with suspected ONJ, as they can detect early changes in the trabecular and cortical bones [2]. Furthermore, 2-(18F)-fluoro-2-deoxy-D-glucose position emission tomography (FDG-PET) is one of the most effective techniques to detect and evaluate the severity of ARONJ [3]. In a systematic review on the development of BRONJ, Khan et al. [4] identified infection, administration of BP, tooth extraction, and underlying anemia as risk factors. A 2017 position paper by the Japanese Allied Committee on Osteonecrosis of the Jaw stated that surgical stresses, such as extraction, dental implant surgery, and periodontal surgery, were considered as local risk factors, while steroid treatment, diabetes, malignant tumors, cancer chemotherapy, smoking, and poor oral hygiene were considered as systemic risk factors [5].

To date, studies on the imaging modalities and risk factors of ONJ have focused on assessing its stage and predicting its development, and factors associated with refractory disease or an exacerbation of ONJ have not been investigated in detail. The only predictive data on surgical outcomes are those reported by Fleisher et al. [6, 7] using FDG-PET. It is important to understand these factors in detail to treat ARONJ successfully.

This study aimed to predict the possibility of treatment resistance among ARONJ patients based on clinical and imaging factors.

## Patients and methods

This study was approved by the institutional review board of our hospital (No. IRB 2018–149).

### Treatment protocol for stage I and II ARONJ

**Fig. 1:**
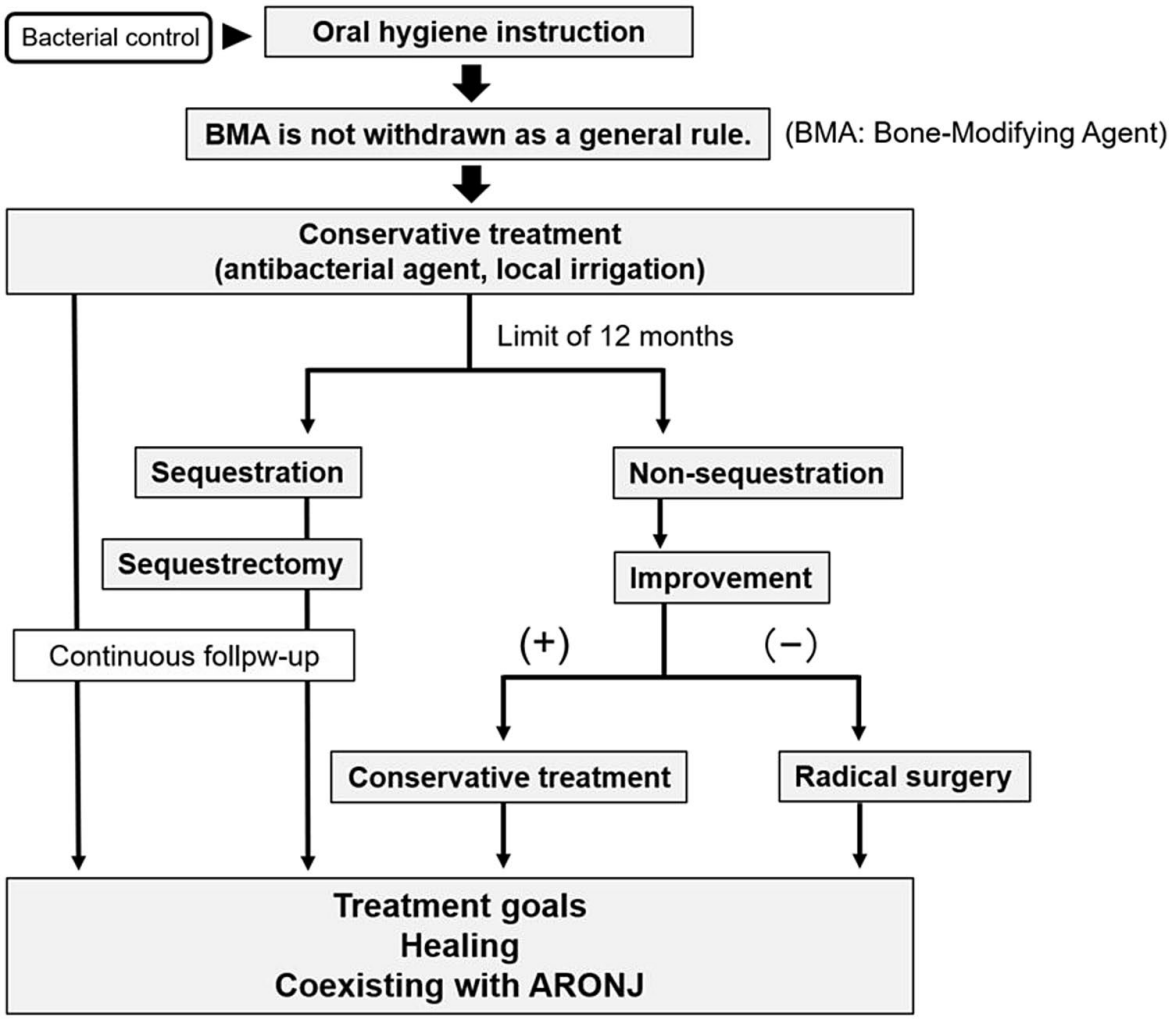
Treatment protocol for stage I and II ARONJ at our institution. The purpose of our treatment protocol was to prevent the progression of osteonecrosis to osteomyelitis of the jaw and maintain the quality of life of ARONJ patients. *ARONJ* anti-resorptive agent-related osteonecrosis of the jaw, *BMA* bone-modifying agent

The treatment protocol for stages I and II ARONJ at our institution is described in Fig. 1 [8]. We included 58 patients with ARONJ who underwent treatment under this protocol over 9 years (January 2009 to December 2017) (Table 1). The main purpose of this protocol was to prevent the progression of osteonecrosis into osteomyelitis and preserve the patients' quality of life. The approach was divided into conservative treatment and radical surgery. Conservative treatment included administration of oral or intravenous antibacterial drugs, antimicrobial mouthwash, local irrigation, oral hygiene management by educating the patient, and sequestrectomy or curettage of the necrotic bone. Sequestrum formation involves pathological encapsulation during the wound healing process, and it is a healing mechanism induced by a normal foreign body reaction in the bone; accordingly, sequestrectomy is classified as a conservative treatment [9]. Conservative treatment was performed for a maximum of approximately 12 months. Radical surgery was of two types: (1) marginal shave/resection, wherein the surrounding bone, including the necrotic part, was extensively resected until the fresh bone was exposed macroscopically; and (2) segmental resection, wherein the necrotic part was resected with a safe region including the healthy bone. There were four possible treatment outcomes: (1) Healing occurred when objective and subjective symptoms (such as bone exposure and infection) disappeared after the therapeutic intervention and the bone was covered by the epithelium; (2) Stage-down corresponded to the observation of objective and subjective findings that were reduced or down-staged; (3) Stable denoted no change in the disease stage or clinical findings after the treatment; and (4) Stage-up indicated the progression of the disease to an advanced stage.

**Table 1:** Patients background

Age/years (median)	68 (50–98)
Administration period/days (median)	1251 (301–2358)
	Cases
Gender	
Male	22
Female	36
Location	
Maxilla	21
Mandibular	37
Anterior and premolar	4
Molar	54
Stage at the first consultation	
I	19
II	39
Medication target disease	
Bone metastasis of malignant tumor (Intravenous administration: 37cases)	37
Osteoporosis (Intravenous administration: 6cases)	21
Risk factor (diabetes mellitus, malignancy, chemo-therapy for malignancy, smoking, steroid administration)	
Present	49
Absent	9

In this protocol, previously reported risk factors, such as steroid treatment, diabetes, malignant tumors, cancer chemotherapy, and smoking were not assessed [5]. Moreover, drug withdrawal was also not assessed because of the seriousness of the causative disease, such as osteoporosis and bone metastasis of malignancy [10–15].

### Clinical factors

We evaluated clinical factors, such as age, sex, staging at the time of the first examination, the region of disease development (maxilla or mandible, and anterior/premolar or molar), administered drugs and their administration periods, the target disease that was being treated (i.e., osteoporosis or bone metastasis of the malignant tumor), and risk factors (i.e., steroids treatment, diabetes, malignant tumors, cancer chemotherapy, and smoking). The stage of ARONJ was diagnosed based on a novel diagnostic definition for ARONJ, as proposed by the Japanese Allied Committee (in the position paper published in 2017) [5] and the American Association of Oral and Maxillofacial Surgeons [16]. Regarding administration of BMAs, specific routes of administration are considered important, such as injection for bone metastasis of malignant tumors, and oral drugs for osteoporosis. Denosumab is an injection that it is used at a low dose to treat osteoporosis, and at a high dose to treat bone metastasis of malignant tumors; the treatment of the target disease is the same as the dosage classification of the drug (high and low dose).

### Imaging factors

**Fig. 2:**
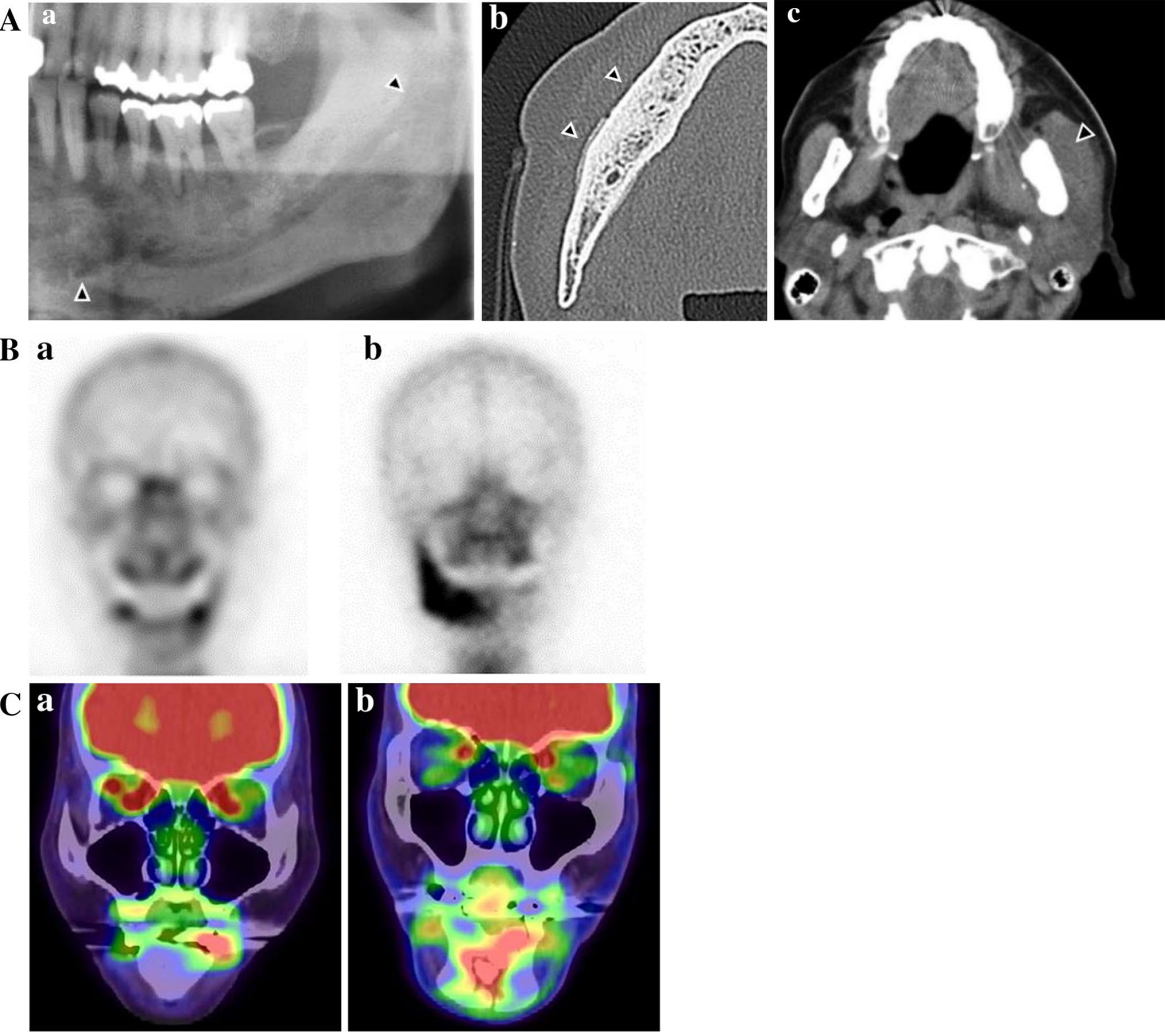
Grading of imaging features. **A** Imaging features of DPR and CT in grading criteria. **a** DPR showing osteosclerotic changes extending into the mandibular canal (black arrow). DPR score 1: Grade 2. **b** CT findings showing a periosteal reaction (black arrow). CT score 2: Grade 1. **c** CT findings indicating the spread of inflammation from the jaw to the masticatory muscles (white arrow). CT score 3: Grade 2. **B** Imaging features of BS in grading criteria. **a** Spot accumulation pattern. BS score: Grade 1. **b** Spread pattern. BS score: Grade 2. **C** Imaging features of FDG-PET/CT in grading criteria. **a** Spot pattern; PET score: Grade 1. **b** Spread pattern; PET score: Grade 2. *BS* bone scintigraphy, *DPR* dental panoramic radiography, *CT* computed tomography, *FDG* 2-(18F)-fluoro-2-deoxy-D-glucose, *PET* position emission tomography

During the first examination, we performed DPR for all 58 patients, and performed CT in 57 patients, bone scintigraphy in 23 patients, and FDG-PET/CT to observe the course of the primary malignant lesion in 15 patients. The DPR and CT images were evaluated as per Obinata et al.’s method [17]. Using these images, we classified osteolysis and osteonecrosis (designated as Score 1) into the following grades: Grade 0 = none; Grade 1 = localized in the alveolar process of the jaw; and Grade 2 = extending beyond the mandibular canal or maxillary sinus. Sequestration, periosteal reaction, and pathological fractures (Score 2) were classified as follows: Grade 0 = not observed; and Grade 1 = observed. CT images that showed disease spread in the soft tissues (Score 3) were additionally classified as follows: Grade 0 = not observed; Grade 1 = localized around the alveolar bone; and Grade 2 = extending into the masticatory muscle, masticatory space, subcutaneous adipose tissue, or maxillary sinus (Fig. 2A; Table 2). The bone scintigraphy (BS) score was defined as follows: Grade 0 = absent; Grade 1 = spot; and Grade 2 = spread (Fig. 2B; Table 2). ARONJ-induced changes in regions showing changes in bone metabolism were based on the PET/CT uptake score as follows: Grade 0 = absent; Grade 1 = spot; and Grade 2 = spread (Fig. 2C; Table 2). We also calculated the maximum standardized uptake value (SUV_max_). The aforementioned grades were determined by two board-certified experts (an oral and maxillofacial surgeon and an oral radiologist) who were blinded to the clinical details of each patient.

**Table 2:** Criteria of imaging diagnosis (grading)

DPR/CT score				
Score 1	Osteolysis and osteoscleorosis (DPR/CT)	Grade	0	Not observed
			1	Located in alveolar bone
			2	Extended into mandibular canal/ maxillary sinus
Score 2	Sequester, periosteal reaction and pathological fracture (DPR/CT)	Grade	0	Absent
			1	Present
Score3	Spread into soft tissue (CT)	Grade	0	Not observed
			1	Spread into alveolar
			2	Spread into masticator muscles or subcutaneous adipose tissue or maxillary sinus
Bone scintigraphy(BS) score, FDG-PET/CT(FDG) score				
Accumulation pattern		Grade	0	Absent
			1	Spot
			2	Spread

### Statistical analysis

First, we performed a power analysis to assess the appropriateness of the sample size by G*Power software (Universität Düsseldorf, Düsseldorf, Germany).

Next, concerning the objective variables, we defined the healing, stage-down, and stable stages as successful outcomes, and the stage-up stage as resistant. To obtain explanatory variables, we determined the cut-off values for the continuous variables of age (68 years), drug administration period (1251 days), and SUV_max_ (6.92) using receiver operating characteristic curves and converted them to binary variables. A DPR score of 1, CT scores of 3, and the BS and PET scores were converted to binary variables of Grade 0, 1 vs. Grade 2, Grade 0 and 1 vs. Grade 2, and absent and spot vs. spread, respectively. Unpaired between-group comparisons were performed for all explanatory variables using the *χ*^2^ test for independence. For significantly different explanatory variables, the odds ratio (OR) was calculated. Furthermore, we examined stage-down as an improvement outcome to compare with the stable and stage-up stages, which were considered as no-improvement outcomes, using the *χ*^2^ test for independence.

Statistical analyses were performed using SPSS software for Windows (version 25; IBM Corp., Armonk, NY). A *p* value < 0.05 was considered significant.

### Imaging analysis

CT was performed using a SOMATOM Definition Flash (Siemens Healthcare Co. Ltd., Forchheim, Germany) and a Light Speed VCT (GE Healthcare Co. Ltd., Chicago, IL, USA). For BS, a low-energy and high-resolution E. CAM scintigraphy apparatus (Canon Co., Ltd., Tochigi, Japan) was used, with technetium-^99m^hydroxymethylene diphosphonate (^99m^Tc-HMDP; Nihon Medi-Physics Co., Ltd., Tokyo, Japan) and technetium-^99m^methylene diphosphonate (^99m^Tc-MDP; Fujifilm Toyama Chemical Co. Ltd., Tokyo, Japan) injected at 740 MBq in each patient. The matrix size was set at 256 × 25 pixels, and the uptake time was 240 s × 2. For PET, 4 Mbq/kg of FDG was intravenously administered after the patients had fasted for at least 6 h. After 60 min, PET images were acquired in a 700-mm visual field using a scanner with 3.27-mm slice thickness (Discovery STE, GE Healthcare). Three-dimensional data were collected at 3 min/bed position, followed by image reconstruction using the 3D-OSEM method. Segmented attenuation correction was applied using X-ray CT (140 kV, 120–240 mAs) and 128 × 128 matrix images were prepared.

## Results

### Patient background

The patients’ background characteristics are presented in Table 1. The patients’ age ranged from 23–93 years, with a median age of 68 years. There were 22 male (37.9%) and 36 female participants (62.1%). ARONJ developed in the maxilla, mandible, anterior and premolar tooth region, and molar region in 21 (33.9%), 37 (66.1%), four (6.9%), and 57 (93.1%) cases, respectively. The stage at the time of the first examination was I and II in 19 and 39 cases, respectively. The target disease was bone metastasis of malignant tumor in 37 cases and osteoporosis in 21. The drug was intravenously administered to all 37 patients with malignancy and in five out of 21 patients with osteoporosis.

### Treatment outcome

The treatment was successful in 53 (91.4%) of the 58 patients, but the disease was resistant in five patients (8.6%). The period of observation was 1 year after the start of the treatment.

### Analysis of factors associated with the refractory cases

At first analysis, regarding the objective variables, we defined the healing, stage-down, and stable stages as successful outcomes, and the stage-up stage as resistant-to-treatment. Considering the clinical factors, the refractory cases had a significantly higher BMA administration period (cut-off: 1251 days; *p* = 0.032; OR: 11.2; 95% confidence interval [CI] 1.115–122.518), and a tendency for high-dose drug administration for bone metastasis of malignant tumors (*p* = 0.102). No significant differences were observed for imaging factors; however, refractory cases tended to exhibit a spreading pattern (BS score: Grade 2; *p* = 0.133) (Table 3). At second analysis, we used the stage-down stage as an improvement outcome to compare with the stable and stage-up stages as no-improvement outcomes. The target disease that was being treated (osteoporosis or bone metastasis of malignant tumor) was the only significant refractory factor (*p* = 0.024, OR: 3.667 95% CI 1.159–11.603), and there were no significant differences among all imaging factors (Table 4).

**Table 3:** Clinical and imaging factors related to treatment outcomes compared *healing*, *stage-down*, and *stable* stages as successful the *stage-up* stage as resistant (Results of univariable analysis with × 2 test for independence)

	Successful	Resistant	*p* value	Odds ratio	95% CI
Clinical factors	(*n* = 57)	(*n* = 5)			
Age					
< 68y	24	1			
≥ 68y	29	4	0.275		
Administration period	(*n* = 38)	(*n* = 5)			
< 1251 days	28	1			
≥ 1251 days	10	4	0.032*	11.2	1.115–112.518
Gender					
Male	20	2			
Female	33	3	0.635		
Stage at the primary consultation					
I	18	1			
II	35	4	0.467		
Location					
Maxilla	20	1			
Mandibular	33	4	0.398		
Anterior and premolar	4	0			
Molar	49	5	0.69		
Medication target diseases					
Bone metastasis of malignant tumor	32	5			
Osteoporosis	21	0	0.095		
Administration route					
Intravenous	38	5			
Oral	15	0	0.21		
Risk factor					
Present	44	5			
Absent	9	0	0.416		
Imaging factors					
DPR	(*n* = 53)	(*n* = 5)			
Score 1					
Grade 0,1	25	1			
Grade 2	28	4	0.248		
Score 2					
Grade 0	46	3			
Grade 1	7	2	0.168		
CT	(*n* = 48)	(*n* = 5)			
Score 1					
Grade 0,1	15	2			
Grade 2	33	3	0.52		
Score 2					
Grade 0	32	2			
Grade 1	16	3	0.239		
Score 3					
Grade 0,1	36	4			
Grade 2	12	1	0.643		
Bone scintigraphy	(*n* = 28)	(*n* = 5)			
Grade 0,1	16	1			
Grade 2	12	4	0.149		
FDG-PET/CT	(*n* = 12)	(*n* = 3)			
Grade 0,1	11	2			
Grade 2	1	1	0.371		
SUV_max_					
< 6.92	10	1			
≥ 6.92	2	2	0.154

**Table 4:** Clinical and imaging factors related to treatment outcomes compared stage-down as improvement with stable and stage-up as no-improvement (Results of univariable analysis with × 2 test for independence)

	Improvement	No-improvement	*p* value	Odds ratio	95% CI
Clinical factors	(*n* = 30)	(*n* = 28)			
Age					
< 68y	12	13			
≥ 68y	18	15	0.621		
Administration period	(*n* = 25)	(*n* = 27)			
< 1251 days	14	16			
≥ 1251 days	11	11	0.812		
Gender					
Male	11	11			
Female	19	17	0.837		
Location					
Maxilla	11	10			
Mandibular	19	18	0.940		
Anterior and premolar	2	2			
Molar	28	26	0.667		
Medication target diseases					
Bone metastasis of malignant tumor	15	22			
Osteoporosis	15	6	0.024*	3.667	1.159–11.603
Administration route					
Intravenous	19	24			
Oral	11	4	0.520		
Risk factor					
Present	23	26			
Absent	7	2	0.089		
Imaging factors					
DPR	(*n* = 30)	(*n* = 28)			
Score 1					
Grade 0,1	14	12			
Grade 2	16	16	0.771		
Score 2					
Grade 0	26	23			
Grade 1	4	5	0.454		
CT	(*n* = 27)	(*n* = 26)			
Score 1					
Grade 0,1	9	8			
Grade 2	18	18	0.842		
Score 2					
Grade 0	18	16			
Grade 1	9	10	0.697		
Score 3					
Grade 0,1	21	19			
Grade 2	6	7	0.691		
Bone scintigraphy	(*n* = 18)	(*n* = 15)			
Grade 0,1	11	6			
Grade 2	7	9	0.227		
FDG-PET/CT	(*n* = 6)	(*n* = 9)			
Grade 0,1	6	7			
Grade 2	0	2	0.371		
SUV_max_					
< 6.92	6	5			
≥ 6.92	0	4	0.092		

### Common factors in the five refractory cases

**Fig. 3:**
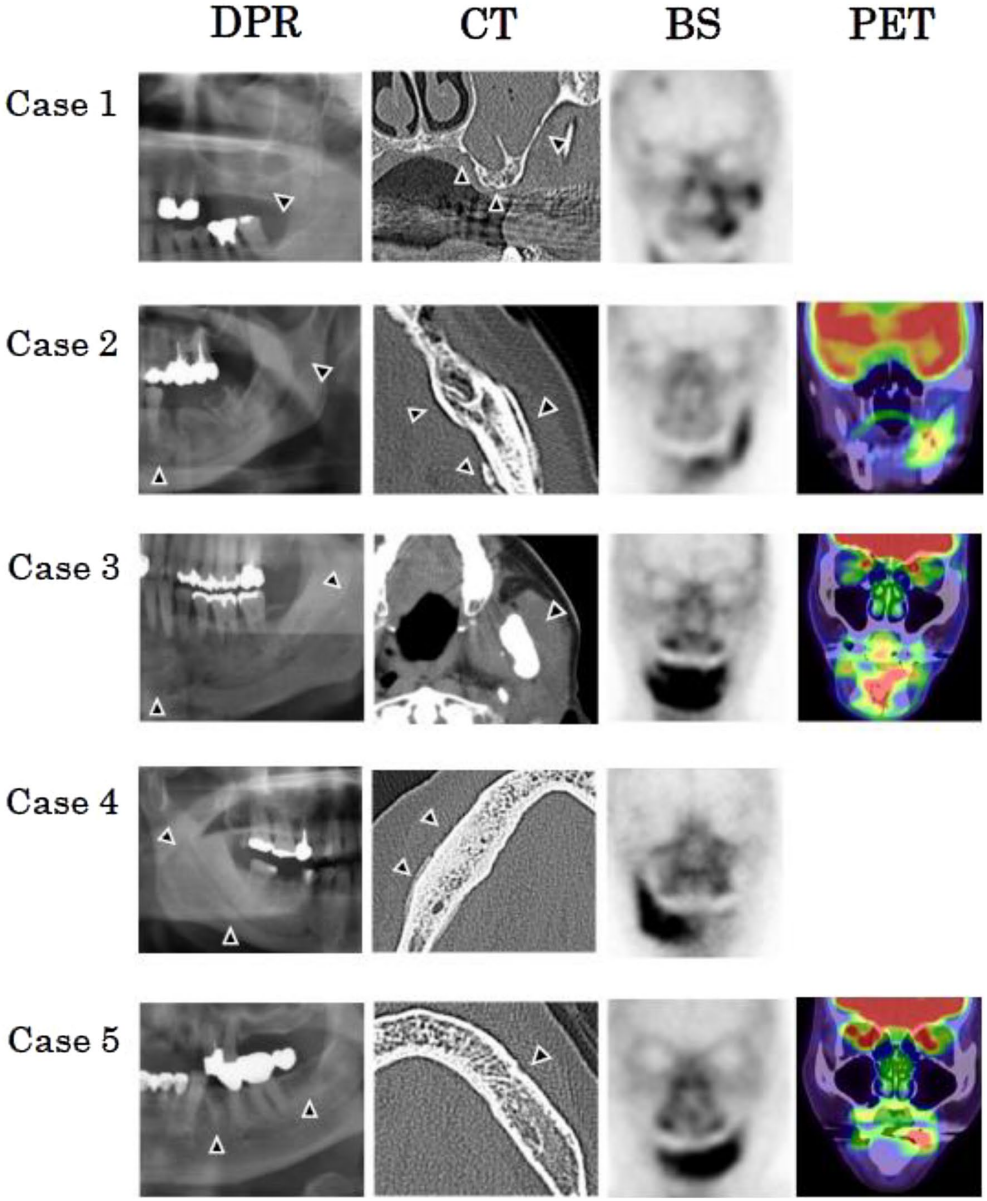
Imaging features in five refractory cases. Patient 1. Location: left molar in the maxilla. **a** DPR: osteosclerotic changes extending to the maxillary sinus (black arrow); DPR score 1: Grade 2. **b** CT findings: osteosclerotic changes extending to the maxillary sinus (black arrow); CT score 1: Grade 2. Bone scintigraphy findings: spot accumulation pattern (black arrow). **c** BS score: Grade 1. Patient 2. Location: left molar region in the mandible. **a** DPR: osteosclerotic changes extending into the mandibular canal (black arrow). DPR score 1: Grade 2. **b** CT findings: osteosclerotic changes extending into the mandibular canal and causing a periosteal reaction (black arrow). CT score 1: Grade 2; CT score 2: Grade 1. **c** Bone scintigraphy findings: spread pattern (black arrow). BS score: Grade 2. FDG-PET/CT: spot pattern. **d** PET score: Grade 1. SUVmax: 4.41. Patient 3. Location: left molar region in the mandible. **a** DPR: osteosclerotic changes extending into the mandibular canal (black arrow). DPR score 1: Grade 2. **b** CT: spread of jaw inflammation to the masticatory muscles (black arrow). CT score 3: Grade 2. **c** Bone scintigraphy: spread pattern (black arrow). BS score: Grade 2. **d** FDG-PET/CT: spread pattern. PET score: Grade 2. SUVmax: 6.92. Patient 4. Location: right molar region in the mandible. **a** DPR: osteosclerotic changes extending into the mandibular canal (black arrow). DPR score 1: Grade 2. **b** CT findings: osteosclerotic changes extending into the mandibular canal and causing a periosteal reaction (white arrow). CT score 1: Grade 2; CT score 2: Grade 1. **c** Bone scintigraphy findings: spread pattern. BS score: Grade 2. Patient 5. Location: left premolar region in the mandible. **a** DPR: osteosclerotic changes in the alveolar bone (black arrow). DPR score 1: Grade 1. **b** CT findings: osteosclerotic changes in the alveolar bone (white arrow). CT score 1: Grade 1. **c** Bone scintigraphy findings: spread pattern. BS score: Grade 2. **d** FDG-PET/CT: spot pattern. PET score: Grade 1. SUVmax: 8.75. *BS* bone scintigraphy, *DPR* dental panoramic radiography, *CT* computed tomography, *FDG* 2-(18F)-fluoro-2-deoxy-D-glucose, *PET* position emission tomography, *SUV*_*max*_ maximum standardized uptake value

We investigated the common factors in the five refractory cases. Among the clinical factors, high-dose drug administration for bone metastasis of malignant tumor was common to all cases, and the disease was located in the mandibular molar region in four out of five cases (Table 5). Two imaging factors, DPR/CT score 1 (osteolysis and osteonecrosis: Grade 2, extension into the mandibular canal/maxillary sinus) and BS score 2 (spread pattern) were common in four out of five cases. An SUV_max_ value of 8.75 was considered to be high in one case, although the PET score of the patient was of Grade 1 (Case 5) (Table 6; Fig. 3).

**Table 5:** Clinical factors of repellant cases

No	Gender	Age	Location	Stage	Administered drug	Administraton period (days)	Diabetes mellitus	Underlying disease	Chemo-therapy for malignancy	Smoking	Steroid
1	F	75	Left molar	2	Denosumab	889	+	Malignancy	+	−	−
			Maxilla								
2	F	69	Left molar	2	Denosumab	1326	−	Malignancy	+	−	−
			Mandibule								
3	M	61	Left molar	2	Denosumab	1304	−	Malignancy	+	−	+
			Mandibule								
4	M	68	Right molar	2	Denosumab	2010	−	Malignancy	−	−	−
			Mandibule								
5	F	74	Left molar	1	Zoledronate	2086	−	Malignancy	−	−	−
			Mandibule

**Table 6:** Imaging factors of repellant cases

No	DPR score1	DPR score2	CT score1	CT score2	CT score3	BS score	FDG score	SUV_max_
1	2	0	2	0	1	2	–	–
2	2	1	2	1	2	1	1	4.41
3	2	1	2	1	1	2	2	6.92
4	2	0	2	1	1	2	–	–
5	1	0	1	0	0	2	1	8.75

## Discussion

Our treatment protocol for stages I and II ARONJ was based on the mechanism and pathophysiology of ARONJ development. In a previous study [8], no background risk factors that influenced the healing of stages I and II ARONJ were identified. Thus, we did not consider risk factors while administering our protocol. In this study, we found that the risk factors were not similar to refractory factors. A BMA administration period of > 3.5 years (1251 days) was the only predictor of treatment resistance. In addition, we did not investigate drug withdrawal during treatment because of the seriousness of the causative diseases. Therefore, the treatment outcome was favorable in 91.4% of stage I and II ARONJ patients, which suggested that the majority of them can be healed and stabilized by infection control and patient education.

Recognizing the differences between osteonecrosis and osteomyelitis is important when considering the mechanism of ARONJ development. Osteonecrosis can be an ischemic and aseptic process that frequently develops in the femoral head and causes localized bone necrosis by blocking its blood supply. This condition transitions into osteomyelitis when a bacterial infection occurs in the necrotic bone; this similarly occurs in the maxillary and mandibular bones. With this background, we hypothesized that the fundamental pathophysiology of ARONJ development is the loss of balance in local bone remodeling due to osteoporosis or bone metastasis of the malignant tumor. Excess suppression of osteoclastic activity due to the long-term use of BMAs inhibits bone resorption, which in turn inhibits bone metabolism and leads to a state of low bone turnover. This condition progresses into metabolic disturbance due to reduced formation of the medullary cavities, which distributed, such as osteosclerosis, in morphological image.

The decreasing blood circulation volume subsequently causes necrosis of the jaw. Prolonged circulatory failure reduces local immunity and makes the necrotic bone susceptible to infections from oral bacteria, which results in osteomyelitis of the jaw. Therefore, we believe that osteomyelitis occurs in a state of metabolic suppression and circulatory failure, where the normal mechanism of wound healing is not triggered, leading to a refractory condition. In a study on the developmental mechanism of BRONJ using diagnostic imaging, Obayashi et al. [18] observed that the increased levels of ^99m^Tc-MDP in the bone were mainly a result of a low local pH, which induced acidosis and increased accumulation of BMAs. Metabolic disturbance was associated with prolonged hypoxic state of the bone and inhibition of the normal mechanism of wound healing, which eventually led to refractory conditions.

In this study, five patients (8.6%) were resistant to our ARONJ treatment protocol. Thus, we examined the possibility of predicting refractory stage I and II ARONJ using the clinical and imaging factors. First, the healing, stage-down, and stable stages were considered as successful outcomes to compare with the stage-up stage, which was considered as resistant-to-treatment. Next, we examined stage-down as an improvement outcome to compare with the stable and stage-up stages, which were considered as no-improvement outcomes. A BMA administration period of 3.5 years was an important predictor of treatment resistance. High-dose treatment of bone metastasis of malignant tumors was common to all patients, and the lesion was located in the mandibular molar region in four out of five patients. Two imaging factors, DPR/CT score 1 and BS score of Grade 2 (spread pattern), were also found in four patients. Based on these findings, we postulated that a patient having a BMA administration period of > 3.5 years, disease development in the mandibular molar region, a target disease that was being treated as bone metastasis of a malignant tumor, morphologically extensive osteosclerotic features, and metabolic abnormalities may develop refractory ARONJ.

Regarding the diagnostic imaging of ARONJ, Fleisher et al. [6, 7] showed that FDG-PET/CT can be used to visualize a limited region with elevated metabolism as it can depict metabolic and morphological changes. Thus, regions with ARONJ-induced metabolic changes that are not detectable on plain radiography can be identified using this method. Therefore, they demonstrated that FDG-PET-CT may also help predict the course of postsurgical healing, as a limited FDG uptake in the alveolus, torus, and/or basal bone superior to the mandibular canal is a predictor of successful healing after marginal resection (positive predictive value = 1.0) [6, 7]. FDG accumulates in the inflammatory cells in the sequestrum or bone marrow on FDG-PET, and this increased blood flow activity is evaluated with ^99m^Tc on BS; this mechanism led Kitagawa et al. [19] to suggest that ^99m^Tc accumulation may indicate an inflammatory reaction. This accumulation mechanism is undoubtedly useful for evaluating the activity and treatment effects of ARONJ [20]. In the five refractory cases examined in this study, the morphological DPR and CT scores were of Grades 0–2 and 0–1, respectively, but a bone metabolic factor, the BS score, was of Grade 2 (spread) and was accompanied by a high SUV_max_ value of 8.75. Thus, evaluating the activity of refractory ARONJ may be difficult by conventional imaging, and it is important to further analyze the metabolic changes using FDG-PET/CT and BS. Cases 2 and 5, which showed a spread pattern on BS also exhibited a spot pattern on FDG-PET/CT. As a difference in the pathology of regions with a metabolic abnormality can be investigated by combining images showing different rates of drug accumulation, it may be possible to use this relationship to predict treatment resistance.

This study had a few limitations that should be acknowledged. First, we performed FDG-PET/CT to detect the metastasis and progression of the primary malignant disease rather than to examine ARONJ. Thus, there was a significant time lag between the timepoints of acquisition of data and the exacerbation of clinical symptoms of ARONJ. Future studies should be performed in a medical care environment, where FDG-PET/CT would be conducted to plan ARONJ treatments. Second, BS was useful for identifying the inflammatory region; however, identifying the inflammatory activity was difficult as there was no objective index to evaluate the accumulated intensity or volume of the inflammation. To solve this issue, we recommend that SPECT/CT be used more frequently to quantitatively analyze the BS score and improve the methods of mapping bone metabolism.

## Conclusion

Our treatment protocol successfully controlled ARONJ in 91.4% of our patients, suggesting that a majority of patients with stage I and II ARONJ can be healed and stabilized by infection control and patient education. Based on this study’s findings, we recommend that the risk of refractory ARONJ should be evaluated in patients who receive BMAs for > 3.5 years, those with lesions in the mandibular molar region, those in whom the target disease is treated as bone metastasis of the malignant tumor, and in those with morphological osteosclerotic findings and bone metabolic abnormalities. We believe that a combination of metabolic imaging modalities, FDG-PET/CT, and BS may be useful for oral surgeons and oral radiologists to evaluate the disease activity and predict the onset and course of refractory ARONJ in the future.
